# Risk factors for breast cancer in young women by oestrogen receptor and progesterone receptor status

**DOI:** 10.1038/sj.bjc.6601293

**Published:** 2003-10-28

**Authors:** M R E McCredie, G S Dite, M C Southey, D J Venter, G G Giles, J L Hopper

**Affiliations:** 1 Department of Preventive and Social Medicine, University of Otago, Dunedin, New Zealand; 2 Centre for Genetic Epidemiology, University of Melbourne, Level 2, 723 Swanston Street, Carlton, Melbourne, Victoria 3053, Australia; 3 Genetic Epidemiology Laboratory, Department of Pathology, University of Melbourne, Melbourne, Victoria, Australia; 4 Murdoch Children's Research Institute, Royal Children's Hospital, Melbourne, Victoria, Australia; 5 Cancer Epidemiology Centre, Cancer Council of Victoria, Melbourne, Victoria, Australia

**Keywords:** breast cancer in young women, epidemiology, oestrogen and progesterone receptor status

## Abstract

We used data from 765 cases and 564 controls in the population-based Australian Breast Cancer Family Study to investigate whether, in women under the age of 40, the profile of risk factors differed between breast cancer subtypes defined by joint oestrogen and progesterone receptor status. As hypothesised, no significant differences were found.

It has been postulated that joint oestrogen (ER) and progesterone receptor (PR) status might define aetiologically distinct subtypes of breast cancer (Potter *et al*, 1995). The ER+PR+ subtype predominates in western countries (64–68% in postmenopausal women) (Thorpe, 1988; Potter *et al*, 1995), but is less common in Japanese women (29%) (Nomura *et al*, 1992). Neoplasms in women with a germline *BRCA1* mutation tend to be ER− (Phillips, 2000). The pattern of age-specific incidence rates, according to the joint ER/PR status, was similar in Danish women up to the age of 43 years, but differed distinctly thereafter (Yasui and Potter, 1999). This suggests that, if hormone receptor status is related to aetiology, such a relationship may be restricted to older women and not be evident in those under the age of 40 years.

Of published reports on ER or PR status and breast cancer risk, only three were population-based studies that examined differences in risk factor profile for the breast cancer subtypes ER+PR+, ER+PR−, ER−PR+, and ER−PR−. One was restricted to postmenopausal women (Potter *et al*, 1995), a second, predominantly to women over the age of 40 years (Huang *et al*, 2000), and the third to women aged 20–44 years (Britton *et al*, 2002). We used data from the Australian Breast Cancer Family Study (ABCFS), a population-based case–control–family study comprising women with breast cancer diagnosed before the age of 40 years and controls, to investigate whether, in that age group, the profile of risk factors differed between breast cancer subtypes defined by joint ER/PR status. Based on the findings in Danish women (Yasui and Potter, 1999), we hypothesised that there would be no difference in this age group.

## MATERIAL AND METHODS

The study was carried out in Melbourne, Victoria, and Sydney, New South Wales, during 1992–1999 (Hopper *et al*, 1994,^1999^; McCredie *et al*, 1998). All participants provided written informed consent prior to participation in the study, which was approved by the human research ethics committees of the University of Melbourne and the Cancer Councils of Victoria and New South Wales.

Cases were identified from the population-based Victorian and New South Wales cancer registries (to which notification of all cancer diagnoses is mandatory), and comprised all women living in the metropolitan areas of Melbourne and Sydney who were aged less than 40 years at the diagnosis of a histologically confirmed first primary cancer of the breast (ICD-9 174). Recruitment began with a letter to the attending doctor, requesting permission to approach the woman. If permission was granted, a letter to the woman sought her participation. Of 1208 eligible cases, 856 (71%) agreed to participate. Reasons for nonparticipation included: death (2% of total eligible); refusal by the attending doctor (8%) or the woman (14%); nonresponse by the attending doctor (1%) or the woman (1%); and the woman having moved and unable to be located (3%).

Potential controls were women aged less than 40 years and living in the metropolitan areas of Melbourne and Sydney, who were selected from the electoral roll (to which adult registration is compulsory in Australia) using proportional random sampling based on the expected age distribution of the cases. Of 913 eligible controls, 600 (66%) agreed to participate, 27% refused and 8% did not respond.

Participation by cases and controls included a face-to-face interview in the subject's home. The two questionnaires covered: (a) demographic and ethnic background; height; weight; medical history; reproductive factors; and use of oral contraceptives, hormone replacement therapy, tobacco, and alcohol and (b) family history of breast and other cancers. In the period 1992–1995, participation was restricted to cases and controls who could speak English, while in the period 1996–1999 non-English speakers were also included.

We obtained both ER and PR status of the tumour for 694 (81%) participating cases (92% of women diagnosed in 1996–1999; 72% of those diagnosed in 1992–1995) – 87 through immunohistochemical testing (described in Armes *et al*, 1999) of tumour tissue held by the ABCFS, 405 from the histopathology report held at the cancer registry, and 202 through a written request to the pathology laboratory that issued the diagnostic histopathology report. ER/PR status was determined using methods that were immunohistochemical (64%), biochemical (34%) or unknown (2%).

For homogeneity, women known to have a deleterious germline mutation in either BRCA1 or BRCA2 were excluded. Germline testing has to date identified 42 cases with a deleterious mutation in either BRCA1 or BRCA2; a full description of the methods and extent of mutation testing can be found in Dite *et al* (2003).

For ease of interpretation, women of ‘Asian descent’ were excluded, as they differ from ‘western’ women not only with respect to their pattern of ER/PR subtypes (see above) but also in the magnitude of their risk of breast cancer. ‘Asian descent’ was defined as having any grandparent with a southeast Asian ethnic background (those reported here were Chinese, Japanese, Malaysian, Vietnamese, Korean, Filipino and Thai); 50 cases and 36 controls were of ‘Asian descent’. One case of ‘Asian descent’ carried a mutation. Thus, 765 cases (618 with known ER/PR status) and 564 controls were used in the analyses below.

### Statistical analysis

Polytomous logistic regression models were used to estimate breast cancer risk in five groups defined by ER/PR status (ER+PR+, ER+PR−, ER−PR+, ER−PR−, and either ER or PR unknown) in relation to the following known or suspected risk factors: family history of breast cancer reported in a first-degree relative (no, yes); height (<163 cm, ⩾163 cm); body mass index (BMI; <23, ⩾23; 1 year before diagnosis (cases) or interview (controls)); age at menarche (<13 years, ⩾13 years); parous (no, yes); number of live births (0, 1, 2, ⩾3); age at first live birth (<25 years, ⩾25 years, for parous women only); and whether oral contraceptives had ever been used (no, yes). Cut-points were chosen to be consistent with Potter *et al* (1995) if feasible or, when this resulted in markedly uneven groups, were based on the distribution in controls. Differences in proportions, means and odds ratios were evaluated by unconditional logistic regression, analysis of variance, and the likelihood ratio test, respectively, using STATA software.

## RESULTS

The mean age at diagnosis of the 765 cases was 34.9 years (s.d. 3.6), with no difference in age between the five groups defined by ER/PR status (*P*=0.3). Among the 564 controls, the mean age at recruitment was 33.7 years (s.d. 4.4). Cases with known and unknown ER/PR status were similar with respect to educational level (*P*=0.4), marital status (*P*=0.5), and whether or not they had been born in Australia (*P*=0.1).

Among the 618 tumours with known ER/PR status, the distribution of ER/PR subtypes was 53% ER+PR+, 6% ER+PR−, 13% ER−PR+, and 29% ER−PR−, and there was no difference in this distribution between tumours diagnosed in 1992–1995 and those diagnosed in 1996–1999 (*P*=0.1).

Table 1

**Table 1:** Risk of breast cancer defined by ER/PR subtype in Australian women under the age of 40 years according to risk factor

	**ER+PR+**		**ER+PR−**		**ER−PR+**		**ER−PR−**		**ER? &/or PR?**		**Control**	
	***N*****=323**	**OR (95% CI)**^c^	***N*****=34**	**OR (95% CI)**	***N*****=80**	**OR (95% CI)**	***N*****=181**	**OR (95% CI)**	***N*****=147**	**OR (95% CI)**	***N*****=564**	**LRT**^b^
*1° relative with breast cancer*												
Yes	9%		12%		8%		11%		9%		4%	
Yes (ref: No)		2.5 (1.4–4.6)		3.4 (1.1–11)		1.8 (0.6–4.8)		3.4 (1.8–6.4)		2.2 (1.1–4.5)		*P*=0.8

*Height*												
⩾163	59%		62%		70%		59%		58%		55%	*P*=0.3
⩾163 (ref: <163)		1.2 (0.9–1.6)		1.3 (0.6–2.7)		1.8 (1.0–3.1)		1.3 (0.9–1.8)		1.4 (0.9–2.1)		

*Body mass index*												
⩾23	43%		50%		46%		50%		51%		46%	*P*=0.5
⩾23 (ref: <23)		0.9 (0.7–1.2)		1.2 (0.6–2.3)		1.0 (0.6–1.6)		1.1 (0.8–1.5)		1.3 (0.9–1.3)		

*Age at menarche*												
⩾13 years	60%		65%		62%		53%		60%		63%	
⩾13 years (ref: <13)		0.8 (0.6–1.1)		0.9 (0.5–1.8)		0.8 (0.5–1.3)		0.6 (0.4–0.9)		0.9 (0.6–1.3)		*P*=0.5
												
*Ever use of oral contraceptives*												
Yes	93%		91%		96%		92%		91%		91%	
Yes (ref: No)		1.1 (0.7–1.9)		1.3 (0.4–4.0)		1.1 (0.5–2.5)		0.9 (0.5–1.6)		1.0 (0.6–1.9)		*P*=0.6

*Ever had live birth*												
Yes	69%		74%		73%		72%		73%		65%	
Yes (ref: No)		1.0 (0.7–1.4)		1.7 (0.7–4.4)		1.4 (0.8–2.6)		1.0 (0.7–1.6)		1.0 (0.6–1.7)		*P*=0.9

*Age at first live birth*^d^												
⩾25	67%		64%		69%		56%		56%		59%	
⩾25 (ref: <25)		1.7 (1.1–2.5)		1.7 (0.7–4.3)		1.3 (0.7–2.5)		0.9 (0.6–1.4)		1.0 (0.6–1.7)		*P*=0.2

*Number of live births*												
1	13%		24%		28%		17%		14%		12%	
1 (ref: 0)		1.1 (0.7–1.7)		2.6 (0.9–7.1)		2.6 (1.3–5.2)		1.3 (0.8–2.3)		1.1 (0.6–2.1)		
2	35%		26%		28%		36%		38%		29%	
2 (ref: 0)		1.0 (0.7–1.5)		1.3 (0.5–3.8)		1.0 (0.5–2.0)		1.0 (0.6–1.7)		1.1 (0.7–1.9)		
⩾3	21%		24%		18%		19%		21%		24%	*P*=0.8
⩾3 (ref: 0)		1.0 (0.5–1.2)		1.4 (0.5–4.3)		0.9 (0.4–1.9)		0.8 (0.4–1.3)		0.8 (0.4–1.4)		

a ^a^Polytomous logistic regression adjusted for study centre, study period, reference age, highest completed education level, country of birth, marital status, affected first-degree relative, height, body mass index, age at menarche, number of live births, and ever used oral contraceptives.

b Likelihood ratio test.

c Odds ratio with 95% confidence interval.

d Parous women only.

gives the proportion of women by risk factor in each ER/PR-defined group, and shows that the odds ratios for breast cancer according to risk factor did not differ between the ER/PR subtypes (0.2<*P*<0.9). The inclusion of women of ‘Asian descent’, and/or those with a BRCA1 or BRCA2 mutation, and the exclusion of women with unknown ER/PR status, made essentially no difference to this finding.

## DISCUSSION

As hypothesised, this population-based study in non-Asian Australian women under the age of 40 years found, with or without excluding cases known to carry a germline mutation in *BRCA1* or *BRCA2*, no evidence that the effects of any of the major established risk factors differ for breast cancers defined by joint ER and PR status. This lack of heterogeneity in disease in young women accords with the analysis of Yasui and Potter (1999), who applied the age-specific distribution of ER/PR subtypes seen in tumours of 3359 cases (of all ages) in the Danish Breast Cancer Cooperative Group to the national Danish age-specific breast cancer incidence rates (age was the only risk factor examined in their analysis).

The Danish data support the suggestion that ER/PR receptor status might define aetiologically distinct subtypes of breast cancer in older women. While two other population-based studies of breast cancer have claimed evidence for some differences in risk factor profile according to joint ER/PR receptor status (Potter *et al*, 1995; Huang *et al*, 2000), they should perhaps be viewed as hypothesis-generating analyses, given that their findings were based on multiple comparisons, had nominal *P*-values of marginal significance, and some comparisons lacked statements about the statistical significance of observed differences in odds ratios. A third study, of early onset disease, found, as we did, no clear support for aetiologically distinct subtypes (Britton *et al*, 2002). Neither *BRCA1* or *BRCA2* mutation carriers nor women of Asian descent had been excluded from these studies.

Our null finding must be tempered by considerations of statistical power – we could have missed modest differences in risk factor profiles. Each of the previous studies (Potter *et al*, 1995; Huang *et al*, 2000; Britton *et al*, 2002) had similarly limited power. To resolve these issues, there is a need for bigger or combined studies with careful consideration of age at diagnosis, ethnic background, and the status of deleterious germline mutations in known susceptibility genes.
